# Characterization of the CD177 interaction with the ANCA antigen proteinase 3

**DOI:** 10.1038/srep43328

**Published:** 2017-02-27

**Authors:** Uwe Jerke, Stephen F. Marino, Oliver Daumke, Ralph Kettritz

**Affiliations:** 1 Experimental and Clinical Research Center, Charité - Universitätsmedizin Berlin, Max Delbrück Center for Molecular Medicine in the Helmholtz Association, Berlin, Germany; 2 Experimental and Clinical Research Center, Charité - Universitätsmedizin Berlin, Max Delbrück Center for Molecular Medicine in the Helmholtz Association, MDC, Berlin, Germany; 3 Nephrology and Intensive Care Medicine, Charité Campus Virchow, Berlin, Germany

## Abstract

Proteinase 3 is a serine protease found in neutrophil granules and on the extracellular neutrophil membrane (mPR3). mPR3 is a major antigen for anti-neutrophil **c**ytoplasmic **a**ntibodies (PR3-ANCAs), autoantibodies causing fatal autoimmune diseases. In most individuals, a subpopulation of neutrophils also produce CD177, proposed to present additional PR3 on the surface, resulting in CD177^neg^/mPR3^low^ and CD177^pos^/mPR3^high^ neutrophil subsets. A positive correlation has been shown between mPR3 abundance, disease incidence, and clinical outcome. We present here a detailed investigation of the PR3:CD177 complex, verifying the interaction, demonstrating the effect of binding on PR3 proteolytic activity and explaining the accessibility of major PR3-ANCA epitopes. We observed high affinity PR3:CD177 complex formation by surface plasmon resonance. Using flow cytometry and a PR3-specific FRET assay, we found that CD177 binding reduced the proteolytic activity of PR3 *in vitro* using purified proteins, in neutrophil degranulation supernatants containing wtPR3 and directly on mPR3^high^ neutrophils and PR3-loaded HEK cells. Finally, CD177^pos^/mPR3^high^ neutrophils showed no migration advantage *in vitro* or *in vivo* when migrating from the blood into the oral cavity. We illuminate details of the PR3:CD177 interaction explaining mPR3 membrane orientation and proteolytic activity with relevance to ANCA activation of the distinct mPR3 neutrophil populations.

Proteinase 3 (PR3) is a neutrophil serine protease (NSP) found in intracellular granules and vesicles, is present on the outer cell membrane, and is released into the extracellular space during neutrophil activation^1^. Enzymatically active PR3 shapes the inflammatory response by processing mediators, matrix proteins, receptors and intracellular substrates^2^,^3^,^4^,^5^,^6^,^7^. PR3 also serves as a major autoantigen in ANCA-associated vasculitis (AAV)^8^,^9^,^10^. These anti-PR3 antibodies (PR3-ANCA) bind and cross-link membrane PR3 (mPR3) causing neutrophil activation^11^,^12^ which then contributes to necrotizing vasculitis. While all neutrophils contain PR3 intracellularly, the mPR3 pattern is remarkable in that two distinct subsets exist, one displaying little (mPR3^low^) and one abundant (mPR3^high^) mPR3^13^. The percentage of the latter ranges from 0–100%, is genetically determined and stable in a given individual, is significantly higher in AAV patients than in healthy controls, responds stronger to PR3-ANCA *in vitro*, and is associated with worse disease outcome^14^,^15^,^16^,^17^,^18^,^19^. Thus, mPR3 abundance is a critical disease factor. In addition to direct membrane interaction^20^,^21^, we have proposed that high mPR3 levels are achieved by PR3 binding to CD177, a GPI-anchored neutrophil-specific membrane receptor also present in neutrophil granules and released into the extracellular space after neutrophil activation^22^. CD177 and PR3 exist in lipid rafts and recruit a larger signaling complex that allows neutrophil activation after PR3-ANCA binding^23^. Because not all neutrophils express CD177, two populations, namely CD177^pos^/mPR3^high^ and CD177^neg^/mPR3^low^, exist. A physical relationship between CD177 and mPR3 was suggested by co-localization, co-precipitation, and co-expression studies^22^,^24^,^25^,^26^,^27^.

A large population of mPR3^high^ neutrophils results in increased responsiveness to PR3-ANCA, and it is also conceivable that mPR3^high^ neutrophils possess increased membrane-associated proteolytic PR3 activity. We investigated the PR3:CD177 interaction on neutrophils and CD177-transfected HEK293 cells, and used recombinant and neutrophil-derived proteins to study direct interactions *in vitro*. We provide details on the PR3:CD177 complex that help to clarify the presentation of mPR3 to anti-PR3 ANCA, the effects of complex formation on the proteolytic activity of PR3 and its consequences for neutrophil migration.

## Results

### mPR3^high^ and mPR3^low^ neutrophils differ significantly in mPR3 levels, but show similar membrane-associated proteolytic PR3 activity

**Figure 1:**
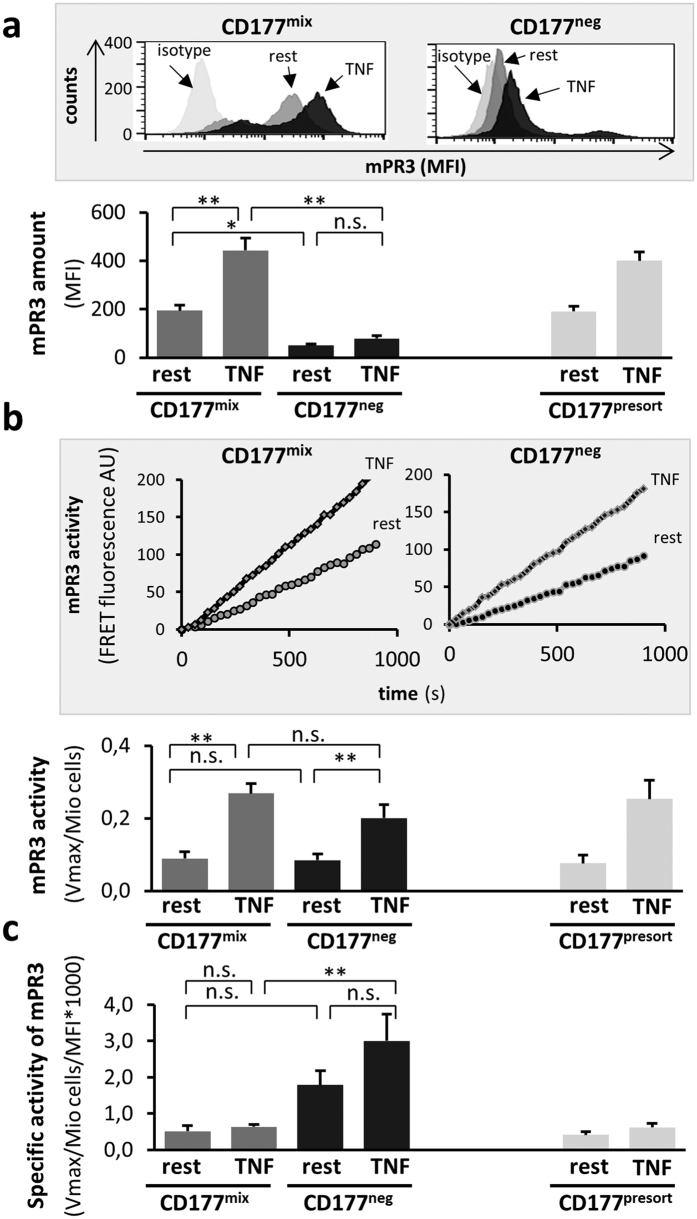
CD177^neg^ and CD177^mix^ neutrophils show different mPR3 levels but similar mPR3 activity. (**a–c**) Pure CD177^mix^/mPR3^mix^ (CD177^mix^) and CD177^neg^/mPR3^low^ (CD177^neg^) neutrophils were obtained from single individuals by magnetic cell sorting. The presort sample is the initial neutrophil preparation that did not go through cell sorting. Neutrophils were treated with buffer control on ice (resting) or with 10 ng/ml TNFα for 15 min at 37 °C (TNFα). (**a**) mPR3 amounts were estimated by flow cytometry (upper panel) and expressed as mean fluorescence intensity (MFI, lower panel). A typical sorting experiment is depicted together with the corresponding statistics. (**b**) After removing degranulated PR3 by washing, the proteolytic mPR3 activity was determined using a PR3-specific FRET assay. (**c**) The specific activity of mPR3 was calculated. Six independent experiments were performed. * indicates p < 0.05 and ** is p < 0.01.

CD177^pos^/mPR3^high^ neutrophils display orders of magnitude more mPR3 than CD177^neg^/mPR3^low^ neutrophils^25^. We first investigated whether or not different mPR3 levels were associated with different mPR3 proteolytic activity on both resting and activated neutrophils. Human CD177^neg^/mPR3^low^ and CD177^mix^/mPR3^mix^ pure neutrophil subsets, depleted of contaminating monocytes and eosinophils, were prepared from single donors by magnetic cell sorting using negative selection. The CD177^mix^/mPR3^mix^ samples contained on average 65% ± 7% CD177^pos^/mPR3^high^ neutrophils. We did not study CD177^pos^/PR3^high^ cells alone, since positive selection with anti-CD177 antibodies results in neutrophil activation. The initial unsorted population (CD177^presort^) was assessed in parallel for comparison. Samples were left resting or were activated with TNFα. After removing degranulated PR3 by washing, we determined mPR3 levels by flow cytometry (Fig. 1a) and mPR3 enzymatic activity using a PR3-specific FRET assay (Fig. 1b). CD177^neg^ resting neutrophils showed little, but detectable, mPR3. TNFα activation caused a measurable, but statistically insignificant increase. Compared to CD177^neg^ cells, CD177^mix^ samples displayed significantly more mPR3 under resting conditions that was significantly increased upon TNFα activation. The FRET assay showed low proteolytic mPR3 activity on resting neutrophils with a significant increase upon TNFα activation. Importantly, resting and activated mPR3 activities were similar between the CD177^neg^ and CD177^mix^ subsets. The results from sorted CD177^mix^ and unsorted CD177^presort^ samples were nearly identical, indicating that the sorting procedure did not influence our results. The calculated average specific PR3 activity was significantly higher on CD177^neg^ than on CD177^mix^ cells (Fig. 1c). Thus, although the mPR3 levels in CD177^neg^ and CD177^mix^ populations substantially differed, the mPR3 proteolytic activity associated with these populations was nearly identical, implying that CD177 binding interferes with PR3 activity.

### CD177 binds PR3 on transfected HEK293 cells and reduces its activity

**Figure 2:**
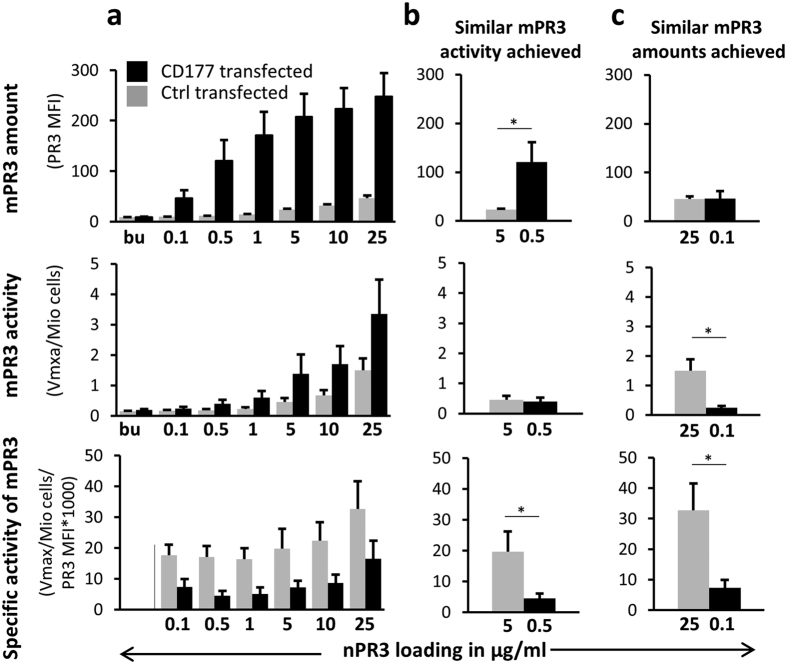
CD177-transfected HEK293 cells bind more PR3 than control-transfected cells, but CD177 binding results in reduced specific activity of mPR3. (**a–c**) HEK293 cells were control (grey) or CD177 transfected (black) and incubated with neutrophil-derived PR3 (nPR3) as indicated. (**a**) CD177-transfected cells presented more mPR3 on their surface and higher mPR3 activity than controls, but showed lower specific activity of mPR3 when incubated with increasing concentrations of nPR3 (n = 6). (**b**) After achieving similar mPR3 activity on control-transfected and CD177-transfected cells by incubating the former with a large PR3 excess, the specific activity of mPR3 was significantly lower on the CD177-transfected cells. (**c**) After achieving similar mPR3 amounts on control-transfected and CD177-transfected cells by incubating the former with a large PR3 excess, the specific activity of mPR3 was again significantly lower on the CD177-transfected cells. * is p < 0.05.

To provide a membrane context free of additional influences that may be present on neutrophils, we repeated our experiments using CD177- and control-transfected HEK293 cells loaded with active PR3. Significantly more PR3 bound to CD177-transfected than to control-transfected HEK293 cells, consistent with our observations with neutrophils (Fig. 2a). The dose-dependent increase in mPR3 resulted in increasing PR3 enzymatic activity. However, the specific activity of mPR3 was significantly higher for the CD177-negative control-transfected cells. We then performed two experiments to further study the specific activity of mPR3 with respect to CD177 binding. First, we achieved similar mPR3 activity on control- and CD177-transfected HEK293 cells by incubation of the former with a ten-fold PR3 excess compared to the latter (Fig. 2b). Although both samples showed similar mPR3 activity, the amount of mPR3 on control-transfected cells was much lower, indicating a significantly higher specific activity in the absence of CD177. Second, we achieved similar mPR3 amounts on both sets of cells (Fig. 2c). This also required incubating the control-transfected cells with a large excess of PR3. Again, control-transfected cells displayed higher mPR3 specific activity than CD177-transfected cells. These data indicate that, although CD177 supports higher mPR3 levels, the resulting PR3 activity is significantly reduced.

### Recombinant CD177 binds PR3 with high affinity and inhibits its proteolytic activity

**Figure 3:**
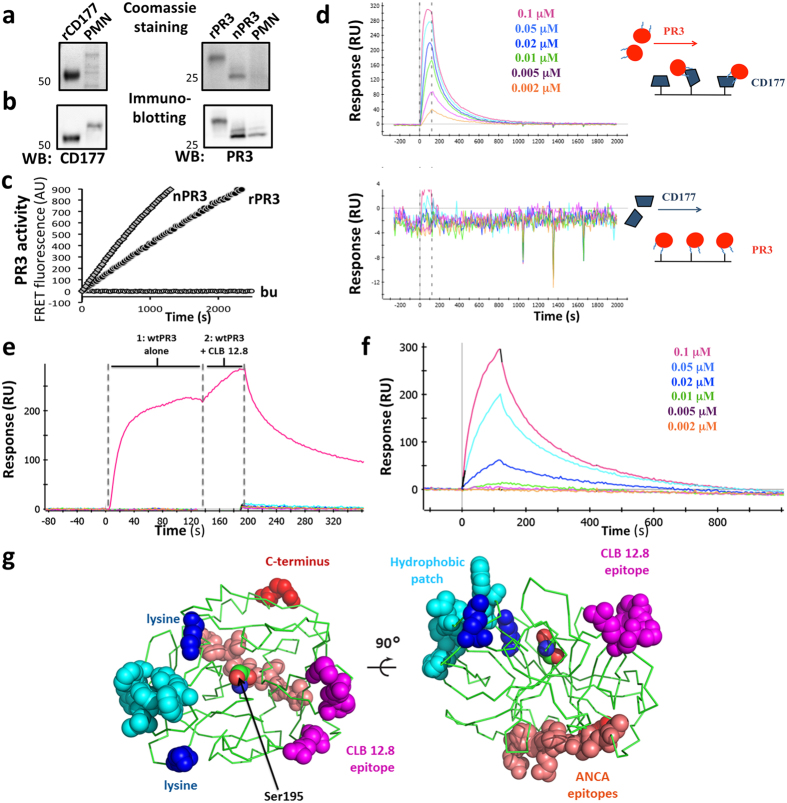
Characterization of rCD177, rPR3 and nPR3, and of the PR3:CD177 interaction. rCD177, rPR3, nPR3, and neutrophil lysates (PMN) were subjected to non-reducing SDS PAGE, (**a**) Coomassie stained and (**b**) immunoblotted. rCD177 lacks the C-terminal GPI-anchor domain and therefore migrates faster than nCD177. rPR3 has 12 additional (assumed unstructured) C-terminal residues that affect migration on the SDS gel compared to nPR3. The molecular weights are indicated by the numbers to the left of each image. (**c**) Enzymatic activity of 1 nM PR3 was measured by FRET assay in the presence of 0.02% lauryl maltoside (LM). (**d**) SPR sensorgrams showing the PR3:CD177 interaction. Vertical dotted lines indicate ligand flow across the chip. Top, CD177 was immobilized to the sensor chip and PR3 at the indicated concentrations was the soluble analyte; high affinity complex formation is observed. Bottom, the same experiment with the reverse configuration: no interaction is observed. Blue lines on the PR3 schematic represent the two lysines available for PR3 immobilization. PR3 is almost certainly uniformly immobilized with its active site facing the chip surface. (**e,f**) Restricting the position of the PR3:CD177 interface. (**e**) SPR sensorgram of the three-way interaction between PR3, CLB 12.8 and CD177. CD177 was immobilized on the sensor chip and subjected to 2 back-to-back ligand flows (horizontal dotted lines). First, soluble PR3 alone was allowed to flow for 120 seconds, producing a resonance units (RUs) increase as PR3 bound CD177; next, a mixture of PR3 and CLB 12.8 was allowed to flow over the chip for 60 seconds and showed an additional RU increase, indicating that CLB 12.8 can bind the complex and, therefore, that the CD177 and CLB 12.8 epitopes do not overlap. (**f**), Sensorgram of the monomeric PR3 hydrophobic patch mutant (Ile217Ala, Trp218Ala) binding to immobilized CD177. (**g**) Orthogonal views of PR3^28^ showing key surface features (rendered using the PyMOL Molecular Graphics System, Version 1.8 Schrödinger, LLC). The backbone is shown in ribbon representation, the hydrophobic patch residues in cyan, the two lysines (Lys) used for immobilization in SPR in blue, the CLB 12.8 epitope residues in magenta, the C-terminal Arg in red and a stretch containing some of the most common human ANCA epitopes^38^ in salmon. The catalytic serine 195 is also shown as spheres.

We next studied direct interactions between CD177 and PR3. We expressed and purified recombinant CD177 (rCD177) and PR3 (rPR3) from 293_6E HEK EBNA cells. These recombinant proteins and neutrophil-derived PR3 (nPR3) showed distinct bands in Coomassie-stained SDS-PAGE, were recognized by specific monoclonal antibodies (mabs), and, for PR3, showed proteolytic activity in the FRET assay (Fig. 3a–c).

By surface plasmon resonance (SPR) spectroscopy, nPR3 and rPR3 showed high-affinity binding with a K_d_ of 4.1 × 10^−9^ M when CD177 was immobilized on the sensor chip. No interaction was observed when the experiment was performed with CD177 as the soluble analyte and PR3 immobilized on the sensor chip (Fig. 3d). To explain the lack of PR3 interaction upon immobilization of PR3, we focused on the lysine side chains in both molecules that were used for immobilization. Although the CD177 structure is not known, we assume its 14 lysine residues are uniformly distributed over the protein’s surface, resulting in multiple orientations on the sensor chip, at least some of which allow interaction with PR3. The X-ray structure of PR3^28^ shows only two lysine residues, flanking the active site on the ‘N-terminal’ face of the protease. This indicates that PR3 is most likely immobilized in a uniform orientation, with its active site facing the sensor chip surface. As this orientation is unproductive for CD177 binding, this observation implies that the CD177 interface is near, or perhaps overlaps, the active site of the protease. To more precisely position the CD177 binding site, we used an anti-PR3 mab (CLB 12.8) whose known epitope is close to the PR3 active site^29^,^30^. By SPR, we observed that PR3:CD177 complexes could bind CLB 12.8 (Fig. 3e), indicating that the CD177 and CLB 12.8 binding sites do not overlap. We also mutated several residues in the surface ‘hydrophobic patch’ of PR3, including two that have been reported^27^ to be important for CD177 binding. These mutations (Ile217Ala, Trp218Ala) produced an active, monomeric PR3 (see below) that bound CD177 with an affinity of 5.7 × 10^−8^ M in SPR – approximately 7-fold lower than wtPR3 (Fig. 3f). This result indicates that the mutated residues are not essential for CD177 binding; their positions, and the CLB 12.8 epitope, are depicted in Fig. 3g.

**Figure 4:**
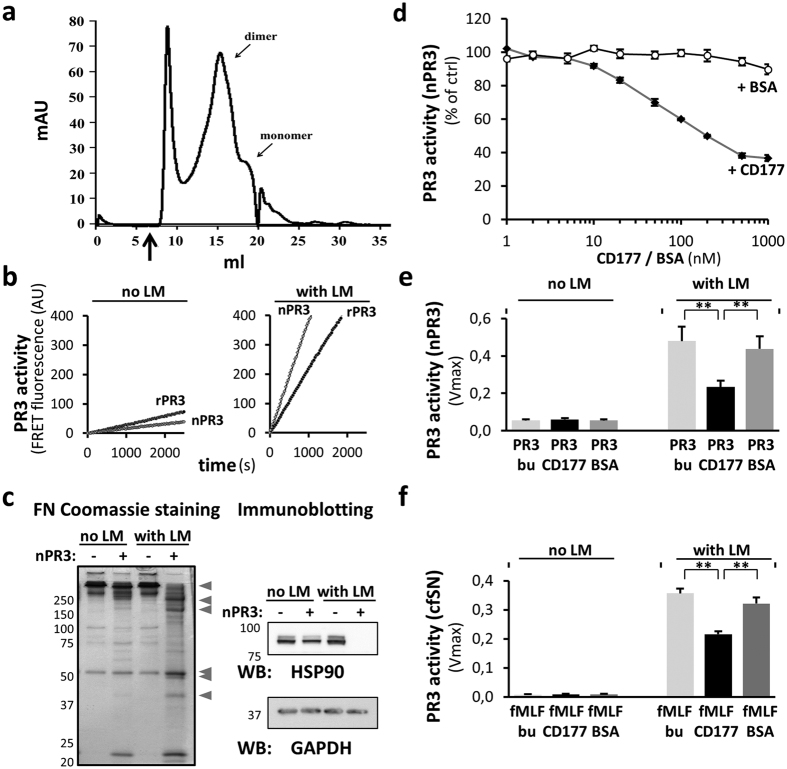
PR3 solubility and activity are increased by detergent. (**a**) S200 size exclusion chromatogram of PR3 in the presence of 0.02% lauryl maltoside (LM). Apparent monomer and dimer species are indicated; the arrow marks the void volume of the column (**b**) Enzymatic activity FRET assays with 1 nM rPR3 and nPR3 in the absence (no) or presence (with) of 0.02% LM. (**c**) nPR3 incubated with fibronectin (FN) or HUVECs protein extract. 16 μg/lane FN was loaded onto a 10% SDS gel and Coomassie stained. Note that several additional FN cleavage products (arrowheads) are seen in the presence of LM. Increased cleavage was also observed by immunoblotting for HSP90 when nPR3 digested HUVECs protein in the presence of LM. (**d**) The effect of rCD177 on the enzymatic nPR3 activity was studied by FRET assay. A dose-dependent activity reduction was observed by incubating 0.5 nM nPR3 with increasing rCD177 concentrations from 1 to 1,000 nM in the presence of 0.02% LM (n = 3). BSA served as control protein. (**e**) The effect of rCD177 on nPR3 activity in the absence and presence of 0.02% LM was studied by FRET assay. 0.5 nM nPR3 was incubated with 50 nM rCD177 or BSA, respectively (n = 5). bu indicates buffer alone control. (**f**) The effect of 50 nM rCD177 on PR3 activity in cell-free supernatants (cfSN) from neutrophils stimulated with 10^−7^ M fMLF was measured in the absence and presence of 0.02% LM by FRET assay (n = 5). ** is p < 0.01.

We observed substantial precipitation of rPR3 *in vitro* under physiological buffer conditions. Addition of 0.02% lauryl maltoside (LM) significantly reduced the irreversible precipitation and facilitated purification. Even in the presence of detergent, rPR3 exists primarily as a collection of high molecular weight species as assessed by size exclusion chromatography, while also showing discernable monomer and dimer fractions (Fig. 4a). LM addition also increased the proteolytic activity of both rPR3 and nPR3 in the FRET assay (Fig. 4b). Moreover, we observed the same activity increase with LM in assays with two natural substrates, fibronectin and HSP90 (Fig. 4c), demonstrating that the LM effect is substrate-independent. We next studied the ability of rCD177 to inhibit PR3 activity. We observed a dose-dependent nPR3 activity reduction upon addition of CD177, but not with bovine serum albumin (BSA) as a control (Fig. 4d,e). The existence of PR3 aggregates was further substantiated by showing that LM also increased PR3 activity in cell-free supernatants (cfSN) released from activated neutrophils (Fig. 4f); this activity could likewise be inhibited by CD177 addition. We conclude from these experiments that wtPR3 readily forms aggregates that interfere with its proteolytic activity and that these effects also occur in biological samples obtained from neutrophils. In addition, CD177 binds to and inhibits the proteolytic activity of PR3 dose-dependently. However, in solution a CD177 excess is needed for this inhibitory effect (see Discussion).

### Supernatants from degranulated CD177^neg^/mPR3^low^ and CD177^mix^/mPR3^mix^ neutrophils contain similar amounts and specific activity of PR3

In addition to association with the neutrophil surface, PR3 is released into the surroundings during neutrophil activation. We assessed PR3 amount and activity in cfSN prepared from CD177^neg^/mPR3^low^ and CD177^mix^/mPR3^mix^ cells sorted from single donors (Supplementary Fig. 1). Cells were left resting or challenged with TNFα or fMLF, after which cfSN were produced. As expected, TNFα degranulated only marginal PR3 amounts, whereas fMLF caused strong PR3 release. Supernatants from resting and stimulated CD177^neg^/mPR3^low^ and CD177^mix^/mPR3^mix^ cells contained, respectively, a similar amount of PR3, with similar specific proteolytic activities despite the presence of CD177 in the mixed population. Western blots of the degranulated supernatants indicated that proportionally more PR3 than CD177 was released from fMLF activated CD177^mix^/mPR3^mix^ cells (Supplementary Fig. 1d,e). The PR3 concentration likely exceeds that of CD177 in this context, resulting in a higher PR3 specific activity for this pool.

### mPR3^high^ and mPR3^low^ neutrophils show similar migration through endothelial cell layers, fibronectin-coated transwells and *in vivo* when migrating into the oral cavity

**Figure 5:**
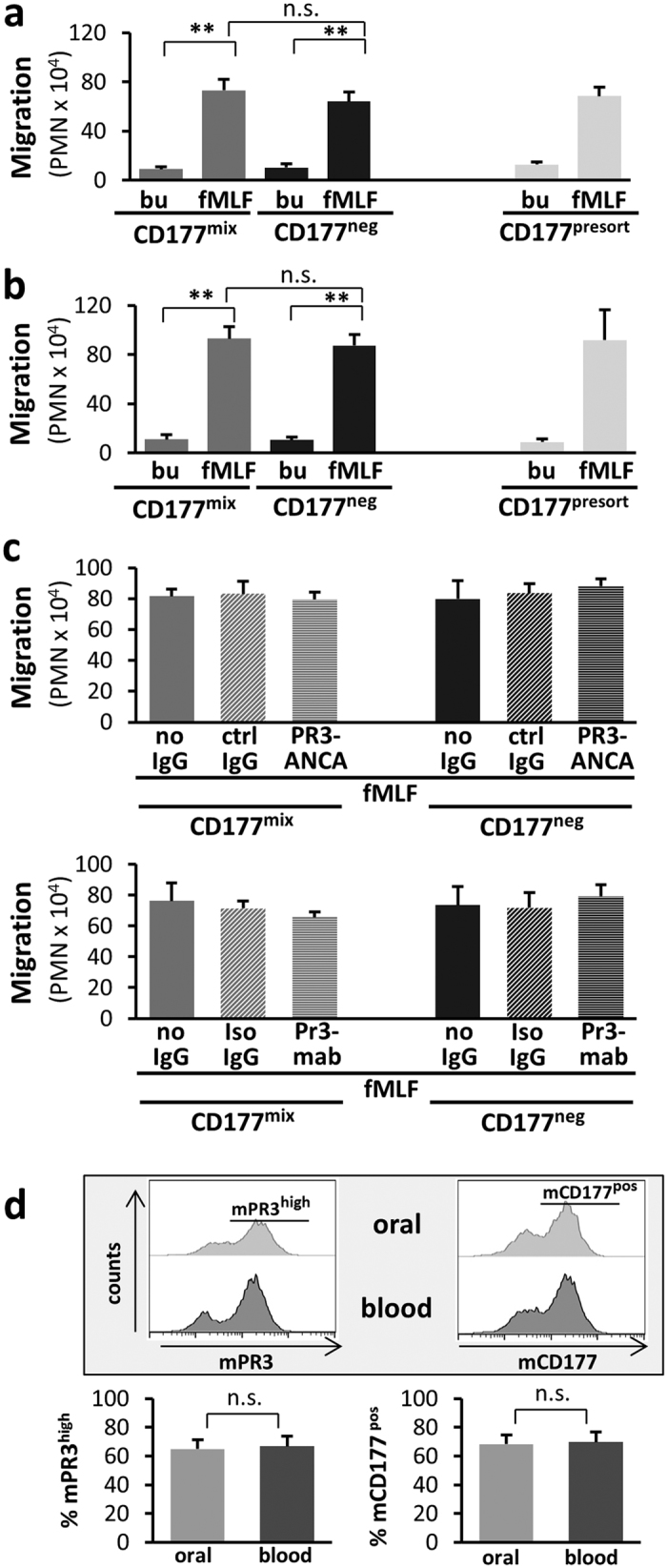
CD177^neg^ and CD177^mix^ neutrophils show similar migration *in vitro* and *in vivo*. (**a–c**) 1.5 × 10^6^ sorted (CD177^mix^ and CD177^neg^) or unsorted (CD177^presort^) neutrophils were added to the upper well and migration towards buffer control (bu) or 10^−8^ M fMLF was assessed. Transmigrated neutrophils (**a**) through fibronectin (n = 4), (**b**) across HUVEC monolayer (n = 4), and (**c**) across glomerular MVEC monolayer in presence of control hu-IgG and PR3-ANCA (upper panel, n = 3) or isotype control and monoclonal anti-PR3 abs (lower panel, n = 3) are shown. ** is p < 0.01. (**d**) Neutrophils of single donors were isolated from the oral cavity (oral) and from blood in parallel (n = 6). The percentage of cells that were either positive for mPR3 or mCD177 was assessed by flow cytometry and was similar in neutrophils from both sites.

It has been assumed that mPR3 activity facilitates neutrophil migration^31^. We therefore assessed migration of CD177^neg^/mPR3^low^ and CD177^mix^/mPR3^mix^ neutrophils in several transwell experiments. When transwells were coated with fibronectin, we found similar migration despite the significant differences in mPR3 levels, consistent with the fact that both neutrophil subsets showed similar PR3 activity on their cell membranes and in degranulated supernatants (Fig. 5a). In order to better account for additional effects potentially present *in vivo*, for example, interaction with cell adhesion molecules, we then performed migration studies using endothelial cell layers. We saw no difference in the migration of the two neutrophil pools across either HUVEC (Human Umbilical Vein Endothelial Cells) (Fig. 5b) or gMVEC (primary human glomerular Microvascular Endothelial Cells) layers, even in the presence of PR3-ANCA (Fig. 5c, upper panel) or a monoclonal anti-PR3 antibody (Fig. 5c, lower panel). Finally, we assessed *in vivo* migration using neutrophils isolated from blood and neutrophils that were harvested from the oral cavity from the same human donor in parallel. We postulated that, if the two CD177/mPR3 neutrophil subsets migrated similarly, the percentage of CD177^pos^/mPR3^high^ neutrophils in blood and mouth rinse would be the same. We indeed found similar percentages of CD177^pos^ and mPR3^high^ neutrophils isolated from both sites (Fig. 5d).

## Discussion

PR3 belongs to the NSP family, but is the only member that also serves as a major autoantigen in AAV. Neutrophils contain abundant PR3 with approximately 3.2 pg per cell^32^. Some is found on the cell membrane of resting neutrophils and significantly more upon activation. Importantly, mPR3 shows a peculiar bimodal pattern^13^ resulting from restricted expression of its GPI-anchored receptor CD177^22^,^24^. The percentage of CD177^pos^/mPR3^high^ neutrophils varies from 0 to 100% in most individuals, whereas approximately 3% of the population lack CD177 expression altogether because of a gene truncation^33^. The clinical significance of a large CD177^pos^/mPR3^high^ neutrophil subset has been demonstrated in AAV^1^,^15^,^16^,^19^,^26^,^34^. It has also been proposed that higher mPR3 protein levels result in higher mPR3 activity and therefore increased matrix degradation and neutrophil migration. In fact, it was suggested that CD177^pos^ neutrophils show higher mPR3 activity than neutrophils from CD177 gene-deficient individuals and that proteolytically active mPR3 facilitates transmigration through endothelial cell layers^31^. However, in that study, neutrophils from CD177 gene-deficient and CD177^mix^ donors showed similar migration through HUVEC coated transwells. Korkmaz *et al*. studied mPR3 activity and found a strong increase upon neutrophil activation; however, this activity increase was independent of the neutrophil population assayed^35^. That is, activated cells from donors with either mPR3^low^ or mPR3^high^ neutrophils showed similar mPR3 activity. Unfortunately, sorted cells were not assessed and variability between donors could have affected the data. We now provide solid evidence that increased mPR3 levels on resting and activated CD177^pos^/mPR3^high^ neutrophils do not show increased mPR3 activity compared to CD177^neg^/mPR3^low^ neutrophils. We excluded inter-individual differences by studying cells from single donors that were sorted into two CD177/mPR3 subsets. Negative cell sorting minimized undesired activation by either anti-CD177 or anti-PR3 antibodies, since both strongly activate neutrophils^23^. We then confirmed our finding using the controlled membrane context of CD177- and control-transfected HEK293 cells.

Based on our neutrophil and HEK293 cell data, we considered the possibility that the association with CD177 was responsible for the lower activity of PR3 on CD177^pos^ cells. We therefore produced recombinant proteins to directly study the PR3:CD177 protein interaction and its effect on the proteolytic activity of PR3. By SPR, we observed high-affinity PR3:CD177 complex formation with a K_d_ of 4.1 × 10^−9^ M, thereby verifying the interaction and extending previous experiments demonstrating PR3 binding to CD177-expressing transfected cells^25^,^27^,^34^. Significantly, we observed that high-affinity CD177 binding reduced the activity of PR3 dose-dependently, thus providing an explanation for the lower specific activity of PR3 observed on CD177^pos^ neutrophils and CD177-transfected HEK293 cells. It is of note that a large stoichiometric excess of rCD177 was required – in all solution experiments – to reach a maximum 50% reduction in PR3 activity. This incomplete inhibition could be related to PR3 forming multimers that may affect accessibility to CD177 though we cannot currently rule out that CD177 recognizes an alternative conformation of the enzyme^36^. Addition of detergent strongly reduced PR3 aggregation and increased PR3’s proteolytic activity towards synthetic and natural substrates. This effect was seen with recombinant protein, with nPR3 and with PR3 degranulated from activated neutrophils.

We also obtained new information concerning the position of the PR3:CD177 interface. The anti-PR3 mab CLB12.8 binds residues Met35, Asn38A, Pro38B and Arg74 (residue numbering from^37^) that cluster near the active site on the opposite side from the hydrophobic patch residues and the lysine residues used for PR3 immobilization to the SPR sensor chips. In SPR, CLB 12.8 and CD177 bound simultaneously to PR3, proving that their epitopes do not overlap. Additionally, a monomeric PR3 hydrophobic patch mutant (Ile217Ala, Trp218Ala) bound CD177 with high affinity, showing that neither these residues nor PR3 multimerization are essential for the interaction. These data help to restrict the location of CD177 binding, which likely occurs near the active site of the molecule. PR3 ‚immobilized’ by CD177 in this orientation on the cell surface also allows solution access to the most common known ANCA epitopes^38^, located on the face of the enzyme opposite the active site. The higher affinity measured for wtPR3 binding is likely due to multivalent interaction of wtPR3 multimers with immobilized CD177.

Finally, when we compared migration of the two separated CD177/mPR3 subsets through fibronectin, HUVEC and gMVEC layers *in vitro*, we observed that migration was independent of mPR3 amount. Moreover, when we studied *in vivo* migration by comparing blood neutrophils with those that had migrated into the oral cavity, we observed again that CD177^pos^/mPR3^high^ neutrophils showed no migration advantage. All of these results are consistent with our finding that the two CD177/mPR3 subsets showed similar mPR3 activities. With the development of more suitable PR3-specific inhibitors, the potential role of mPR3 in migration can be further elucidated.

We conclude that PR3 forms a high-affinity complex with CD177 that is important for its surface presentation as an autoantigen, and also for regulation of its activity as a serine protease. CD177^pos^/mPR3^high^ neutrophils present substantially more PR3 to the extracellular environment, thereby vastly increasing the abundance of ANCA epitopes - but without increasing membrane-bound PR3 activity or affecting neutrophil migration. Given that the increase in ANCA binding sites on CD177^pos^/mPR3^high^ neutrophils correlates with their stronger activation, targeting the PR3:CD177 complex may have therapeutic implications in neutrophil-mediated conditions including PR3-ANCA vasculitis.

## Methods

### Preparation of human neutrophils and cell culture

Blood neutrophils from healthy human donors and human umbilical vein endothelial cells (HUVECs) were obtained according to the operational guidelines of, and after due approval by the Charité (Ethics Votum EA1/277/11). Prior written, informed consent from all subjects was obtained as described previously^5^. Neutrophils from the oral cavity were harvested as described by Ashkenazi^39^. HUVECs were cultured in EBM-2 basal medium, supplemented with EGM-2 SingleQuots (Lonza) and used after two to four passages. Primary Human glomerular Microvascular Endothelial Cells (gMVEC) were obtained from Cell Systems (Kirkland, WA) and cultured in VascuLife basal medium, supplemented with VascuLife VEGF LifeFactors Kit (Lifeline Cell Technology, Frederick, MD).

### Magnetic neutrophil sorting and flow cytometry

Neutrophil subsets were separated with MACS separation columns (Miltenyi Biotec, Bergisch Gladbach, Germany) following the manufacturer’s protocol. The CD177^neg^/mPR3^low^ pure neutrophil subset was negatively selected using anti-CD177 abs (clone MEM166, Exbio, Praha, Czech Republic), anti-CD9 abs for eosinophils (BD Biosciences, San Jose, CA), and anti-CD14 for monocytes (Miltenyi Biotec). The CD177^mix^/mPR3^mix^ pure neutrophil subset was obtained after depletion with anti-CD9 abs and anti-CD14 abs. CD177 and PR3 on neutrophils and HEK293 cells were assessed by flow cytometry as described previously^22^ using 5 μg/ml fluorescence-conjugated mabs to PR3 (clone 40-9-7)^7^ and CD177 (clone MEM166). 10,000 events per sample were assayed using a FACSCalibur (BD Biosciences).

### Neutrophil stimulation and preparation of cell-free supernatants

Neutrophils (1 × 10^7^ cells/ml) were incubated with buffer on ice to prevent degranulation (resting) or activated with 10 ng/ml TNFα (R&D Systems, Wiesbaden-Nordenstedt, Germany) for 15 min at 37 °C. Cells were washed to remove degranulated PR3. mPR3 levels were assessed by flow cytometry and mPR3 activity by FRET assay. For generation of cell-free supernatants (cfSN), 5 × 10^6^ neutrophils were treated with 10 ng/ml TNFα or 5 μg/ml cytochalasin B for 10 min and then incubated with 10^−7^ M f-met-leu-phe (fMLF, Sigma-Aldrich, Deisenhofen, Germany). TNFα treatment results in less neutrophil activation compared to fMLF, which also releases primary granules^40^. After 30 min, samples were centrifuged at 300 g for 10 min to obtain cfSN.

### Assessment of PR3-specific proteolytic activity by FRET

PR3-specific proteolytic activity was measured using the FRET substrate 2-Abz-VAD-norV-ADYQ-EDA-Dnp (Biosynthan, Berlin, Germany) as described^7^. For assessing activity on cells, 1 × 10^6^ neutrophils or 2.5 × 10^5^ HEK293 cells were washed twice, resuspended in in 150 μl HEPES buffer (20 mM HEPES, 150 mM NaCl, pH 7.2) and incubated with 20 μM FRET substrate. For assessing the activity of nPR3, rPR3, and cfSN, 1 nM PR3 or 25 μl cf-SN in 150 μl HEPES buffer were incubated with FRET substrate (20 μM final). Fluorescence was measured by plate reader (excitation 320 nm, emission 420 nm, SpectraMax M5, Molecular Devices, CA) and the corresponding Vmax reported. When indicated the reaction was carried out in 0.02% LM. The specific activity of mPR3 was calculated as Vmax per 10^6^ cells/mean fluorescence intensity for PR3 × 1000 and that of soluble PR3 as Vmax/PR3 in ng/ml × 1000.

### HEK cell transfection with CD177 and PR3 loading

HEK293 cells were cultured and transfected with CD177 as described^41^. CD177 expression was assessed by flow cytometry as described for neutrophils. 2.5 × 10^5^ CD177- or control-transfected cells in 100 μl HBSS were incubated with nPR3-containing buffer (Athens Research&Technology, Athens, GA) between 0.1 and 25 μg/ml as indicated. After 60 min cells were washed twice. mPR3 amounts were determined by flow cytometry and mPR3 activity by FRET assay as described above.

### SDS-PAGE, Coomassie staining, and immunoblot analysis

Cell lysis, SDS-PAGE and immunoblots were performed as described^7^. Immunoblots were developed with a monoclonal rabbit anti-PR3 antibody (Abcam, Cambridge, UK), or the anti-CD177 mab (clone MEM166), polyclonal abs to HSP90 or GAPDH (Santa Cruz, Heidelberg, Germany) as indicated and visualized by enhanced chemiluminescence (Thermo Fisher Scientific).

### Assessment of PR3 by ELISA

PR3 ELISA was used to quantitatively assess PR3 amounts in cfSN from activated neutrophils as described^7^. Briefly, an anti-PR3 capture mab (clone 40-9-7) was coated, blocked and incubated with nPR3 (Athens Research&Technology Inc.) standards or prediluted cfSN (50 μl/well). After washing, biotinylated anti-PR3 detection mab was added. A strepavidin-HRPO conjugate and OPD substrate (Dako, Hamburg, Germany) were used to visualize binding. The absorbance was determined at 405 nm in a plate reader (Molecular Devices).

### Enzymatic PR3 activity towards fibronectin and endothelial HSP90

nPR3 (10 nM final concentration) was incubated with 16 μg fibronectin (FN, Roche, Mannheim, Germany) in 80 μl HEPES buffer for 120 min at 37 °C. 20 μl reaction mix/lane were analyzed via 10% SDS PAGE and Coomassie stained. 15 μg of human umbilical vein endothelial cells (HUVECs) homogenate obtained by mechanical breakup without detergent was incubated with 15 ng nPR3 in 40 μl buffer for 120 min at 37 °C. 20 μl of sample were analyzed via 10% SDS PAGE and immunoblotted for HSP90 and GAPDH.

### Generation of recombinant CD177 and PR3 variants

The proteins were produced from plasmid pTT5 as C-terminal fusions with a human Ig1 Fc, secreted from 293_6E EBNA cells (NRC, Canada) via an N-terminal Ig1 secretion signal peptide (gift of Dr. Felix Oden) and purified from the culture supernatant by passage over immobilized Protein A (UNoSphere, BioRad). Protein A eluted material was immediately neutralized with 1 M HEPES pH 7.5 and Fc removed by addition of TEV protease and incubation overnight at 4 °C. The flowthrough of a subsequent Protein A trap column was concentrated and passed over a Superdex 200 size exclusion column in 20 mM HEPES, 150 mM NaCl, pH 7.5 for CD177 and in the same buffer plus 0.02% LM for PR3 variants. PR3 variants were activated by enterokinase removal of an N-terminal FLAG peptide and passage over anti-M2 agarose (Sigma).

### Surface Plasmon Resonance

Experiments were performed on a ProteOn XPR36 instrument (BioRad) with proteins immobilized to GLH sensor chips (BioRad) using standard amine chemistry.

### PR3 inhibition by CD177

0.5 nM nPR3 or 25 μl of cfSN was incubated with rCD177 or BSA in 150 μl HEPES buffer for 15 min at RT. PR3 activity was measured after addition of 20 μM FRET substrate.

### Neutrophil migration through fibronectin-coated transwells

Migration was tested either in fibronectin-coated transwells or across HUVEC or gMVEC monolayers, (3.0 μm, 6.5 mm from Corning, NY, USA). Neutrophils (1.5 × 10^6^) in HBSS^++^were added to the upper well and stimulated at 37 °C with HBSS or 10^-8^ M fMLF (Sigma) to transmigrate to the lower well. In indicated experiments 100 μg/ml ANCA or control IgGs were also added to the upper well. After 90 min (for FN coating) or 3 hrs (for EC monolayers) transmigrated cells were quantified by MPO assay using a standard curve as described^19^.

### Statistical Analysis

Results are given as mean ± SEM. Comparisons between multiple groups were done using ANOVA and appropriate post-hoc tests. Comparisons between two groups were by two-sided paired t-test. Differences were considered significant if p < 0.05.

## Additional Information

**How to cite this article**: Jerke, U. *et al*. Characterization of the CD177 interaction with the ANCA antigen proteinase 3. *Sci. Rep.* **7**, 43328; doi: 10.1038/srep43328 (2017).

**Publisher's note:** Springer Nature remains neutral with regard to jurisdictional claims in published maps and institutional affiliations.

## Supplementary Material

Supplementary Figure 1
