# Supplementary material for: Characterization of the CD177 interaction with the ANCA antigen proteinase 3
Source: Sci Rep. 2017 Feb 27;7:43328. doi: 10.1038/srep43328 (PMC5327412; doi:10.1038/srep43328)
Supplement: Supplementary Figure 1 [file srep43328-s1.pdf]

Supplementary Information for:

Characterization of the CD177 interaction with the ANCA antigen proteinase 3

Uwe Jerke[1*], Stephen F. Marino[2*†], Oliver Daumke[2] and Ralph Kettritz[1,3†]

[1]Experimental and Clinical Research Center, Charité - Universitätsmedizin Berlin, Max Delbrück Center for Molecular Medicine in the Helmholtz Association, [2]MDC, Berlin, Germany, [3]Nephrology and Intensive Care Medicine, Charité Campus Virchow, Berlin, Germany

* U.J and S.M. share the first authorship and †S.M. and R.K. are corresponding authors

Correspondence to:

Ralph Kettritz, Lindenberger Weg 80, 13125 Berlin, Germany

Tel: +49 30 450540002, Fax: +49 30 450540900,

Email: kettritz@charite.de

and (present address):

Stephen F. Marino, Lindenberger Weg 80, 13125 Berlin, Germany

Tel: +49 30 450540284, Fax: +49 30 450540900

Email: stephen.marino@charite.de

Supplementary Figure 1

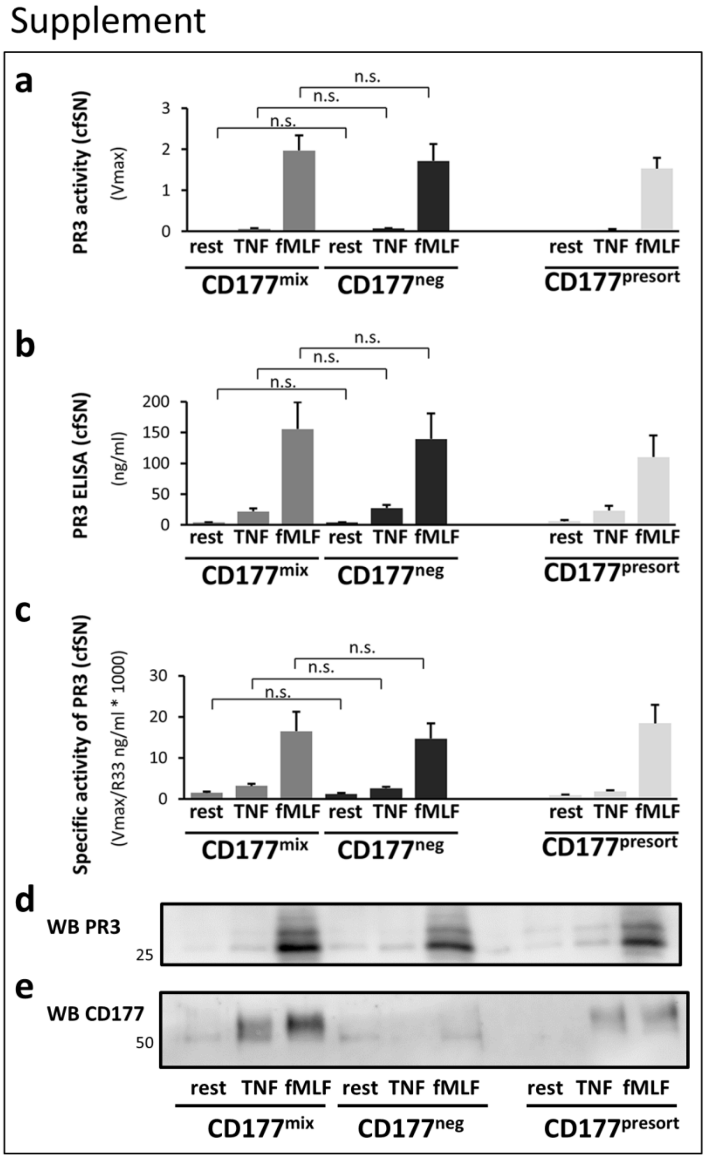

Supplementary Figure 1. Supernatants from CD177^{neg} and CD177^{mix} neutrophils show similar PR3 amounts and activity. Neutrophils were sorted into CD177^{mix} and CD177^{neg} subsets and treated either with buffer on ice (resting), with 10 ng/ml TNFα (TNF) or with cytochalasin B/10^{-7} M fMLF (fMLF) at 37°C (n=5). The CD177^{presort} sample is the initial neutrophil preparation that did not go through cell sorting. After 30 min., cell-free supernatants (cfSN) were generated and (a) the PR3

activity was assessed by FRET assay, (b) the PR3 amount by ELISA, (c) the specific activity of PR3 was calculated from both values. cfSN were assessed by immunoblotting for the presence of (d) PR3 and (e) CD177 to explain the difference in PR3 specific activity between the two different stimuli. While fMLF results in a > 6-fold increase in the pool abundance of PR3 there is no correspondingly large CD177 increase. Either CD177 does not bind PR3 in these supernatants or its binding is saturated due to a large excess of PR3, accounting for the greater specific activity measured for this pool. Numbers to the left of the blots in d and e indicate molecular weights.
